# High-grade B-cell lymphoma manifested as peripheral nerve injury: A case report

**DOI:** 10.1097/MD.0000000000041097

**Published:** 2024-12-27

**Authors:** Han Luo, Shanshan Li, Bo Liu

**Affiliations:** a Department of Neurology, Shenzhen Longhua District Central Hospital, Shenzhen, Guangdong, China.

**Keywords:** chronic inflammatory demyelinating polyneuropathy, high-grade B-cell lymphoma, organomegaly, paraneoplastic neuropathy, peripheral nerve injury

## Abstract

**Rationale:**

High-grade B-cell lymphoma is highly malignant and progresses rapidly, often being at the intermediate or advanced stage with poor prognosis when detected. This disease involves the central nervous system in 9% to 45% of cases, while peripheral nerve injury is relatively rare.

**Patient concerns:**

A 42-year-old male was admitted to the hospital due to recurrent peripheral facial paralysis lasting for 8 months and weakness in both lower extremities lasting for 1 month. No other symptoms and signs were apparent.

**Diagnoses:**

Fluorescence in situ hybridization showed the following results: IgH/Bcl2 t(14:18)(q32;q21) chromosomal translocation: negative; Bc16 gene translocation: positive; and Myc(8;q24) chromosomal translocation: positive. The clonal gene rearrangement test for B-cell lymphoma was positive, and the clonal gene rearrangement test for T-cell lymphoma was negative.The patient was diagnosed with high-grade B-cell lymphoma.

**Interventions:**

The treatment plan included chemotherapy, targeted drug therapy, biological therapy, immunotherapy, etc.

**Outcomes:**

The patient who was followed up for 1 and 3 years had stable conditions and was able to take care of himself, with an mRS score of 1. Five years after the initial diagnosis, the patient experienced recurrence and systemic metastasis of high-grade B-cell lymphoma, ultimately dying from multiple organ failure.

**Lessons:**

Lymphoma manifests mainly as progressive impairment of multiple cranial nerves or recurrent alternating peripheral nerve injury complicated with elevated protein concentrations in cerebrospinal fluid; it can be easily misdiagnosed as chronic inflammatory demyelinating polyradiculoneuropathy. Performing lymph node biopsy for high-grade B-cell lymphoma as early as possible helps detect lymphoma in the early stage.

## 1. Introduction

Central nerve involvement occurs in 9% to 45% of patients with high-grade B-cell lymphoma,^[1–3]^ whereas peripheral nerve injury is rare. Moreover, the diagnosis of peripheral nerve injury is very difficult because of its atypical clinical manifestations and lack of characteristic laboratory and imaging examination indicators. Multiple cranial nerve injuries and elevated cerebrospinal fluid protein levels are rare but important clinical manifestations of high-grade B-cell lymphoma involving the nervous system. We describe 1 case of high-grade B-cell lymphoma with peripheral nerve injury as the initial manifestation to provide new insights into the early diagnosis and treatment of this disease.

## 2. Case presentation

A 42-year-old male was admitted to the hospital due to recurrent peripheral facial paralysis for 8 months and weakness in both lower extremities for 1 month. The patient experienced right facial paralysis after catching a cold 8 months prior, presenting with right eyelid closure weakness, a deviated mouth, water leakage at the right corner of the mouth while gargling and drinking, and a throbbing headache in the right temporal region. The patient was diagnosed with “right peripheral facial paralysis” in a hospital and treated with oral mecobalamin, prednisone, and physiotherapy, and symptoms improved. His facial paralysis had basically recovered 3 months after the treatments. The patient experienced left eye closure weakness and a deviated mouth with no obvious cause, as well as no pain behind the ears 4 months prior. He was diagnosed with “left peripheral facial paralysis” in a hospital and improved after 2 months of treatment with mecobalamin and prednisone. He had limited movement of the left eye and double vision without obvious eye pain. One month prior, the patient experienced weakness in both lower extremities with no obvious cause, causing a feeling of stepping on cotton while walking. His head magnetic resonance imaging (MRI) examination at another hospital revealed “slight ischemia of the bilateral frontal lobes.” After prednisone administration, the patient’s limited movement of the left eye and double vision dramatically improved, with slight double vision remaining when looking downward, and there was no significant improvement in the degree of weakness of either lower extremity. Three days prior, the patient exhibited a deviated mouth to the left after waking in the morning and then gradually experienced poor closure of the right eye, dribbling at the right corner of the mouth and other manifestations. He was admitted to our department for further diagnosis and treatment. The patient experienced paroxysmal limb muscle twitching of varied duration during the course of the disease, with no muscular soreness, general arthralgia, rash, or oral ulcers. He also experienced a 5 kg decrease in body weight in the past month. The patient was previously diagnosed with “chronic superficial gastritis with erosion,” which was subsequently treated with mosapride, ilaprazole and polyethylene glycol. He also had a history of “chronic left epididymo-orchitis.” Physical examination revealed a soft abdomen and impalpable liver and spleen below the costal margin. Examination of the nervous system revealed binocular nystagmus (+), a right eyelash sign (+), and a shallow right nasolabial fold. The patient had grade V-muscle strength in the right lower extremity and normal muscle strength in the remaining extremities. The radioperiosteal reflex in the right upper extremity was impaired, the tendon reflex in the left upper extremity was normal, and the bilateral knee reflex was impaired. Pathological signs were not elicited in either lower extremity. Slightly reduced superficial sensation in the bilateral plantar region and normal sensation and coordinated movement in the remaining regions were observed.

Auxiliary examination: a positron emission tomography-computed tomography (PET–CT) scan performed at the General Hospital of Chinese People’s Armed Police Force Border Defense Force 6 months previously revealed the following: streak-shaped hypermetabolic foci in the bilateral scrota highly indicative of inflammatory lesions; stripe-like hypermetabolic foci in the pericardium and epigastric peritoneum; thickening of nodular soft tissue shadows in the bilateral adrenal glands; inflammatory lesions in the duodenum; increased nodular radiotracer uptake in the right superior alveolus, indicating inflammation; and multiple enlarged lymph nodes in the bilateral submandibular and inguinal regions and below the bilateral sternocleidomastoid muscles. Inflammation was suspected.

**Figure 1.**
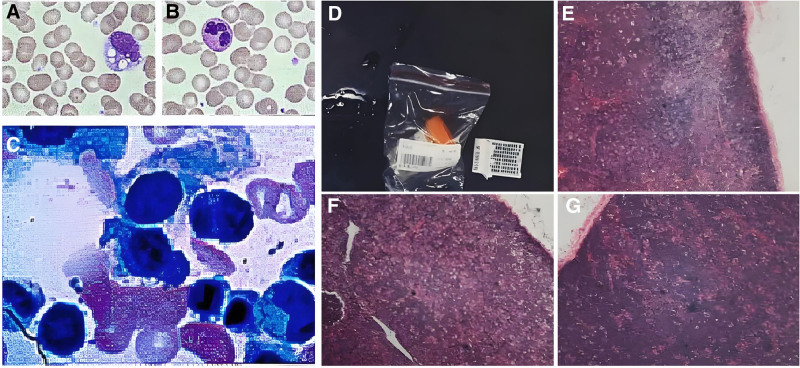
(A, B) In the peripheral blood smear, the morphology of the granulocytes, lymphocytes and platelets was basically normal. Mild anisocytosis was observed in some mature erythrocytes. Some monocytes had intracytoplasmic vacuoles. No nucleated erythrocytes, plasmodium, or microfilaria were observed. (C) Bone marrow puncture revealed the following findings: active bone marrow hyperplasia; hyperplasia of granulocytes, erythrocytes and megakaryocytes; slightly high levels of plasma cells; and individual atypical lymphocytes. (D–G) Lymph node histopathology revealed high-grade B-cell lymphoma, which was consistent with double-hit lymphoma.

A non-contrast brain MRI scan at the First Affiliated Hospital, Sun Yat-sen University, 2 months prior revealed slight ischemia in the bilateral frontal lobes. Color ultrasound at the First Affiliated Hospital, Sun Yat-sen University 2 months prior revealed a hypoechoic lesion in the left testicle with abundant blood supply, a high probability of inflammation, and a cyst in the head of the right epididymis; no abnormalities of the right testicle or left epididymis were found on ultrasound. Electronic gastroscopy at Shenzhen People’s Hospital 1 month previously suggested chronic superficial gastritis with erosion. Pathology revealed chronic active inflammation in the gastric antrum. No significant abnormalities were detected via routine blood tests, routine urine tests, routine stool tests, coagulation panels (4 items), thyroid panels (3 items), erythrocyte sedimentation rate, fasting blood glucose levels, glycosylated hemoglobin levels, renal function tests, myocardial damage, C-reactive protein levels, electrolyte levels, or liver function examinations performed upon admission. Vitamin B12 was 917 ↑ pmol/L, uric acid was 450 ↑ µmol/L, total blood cholesterol was 2.60 ↓ mmol/L, high-density lipoprotein was 0.77 ↓ mmol/L, and low-density lipoprotein was 1.44 ↓ mmol/L. No obvious abnormalities were detected in the urine Bence-Jones protein or chorionic gonadotropin. Immunoglobulin and complement: β2-microglobulin 2.65 ↑ mg/L. Among tumor markers, no significant abnormalities in alpha-fetoprotein, carcinoembryonic antigen, CA125, CA19-9, CA153, CA72-4, total prostate-specific antigen or free prostate-specific antigen were detected. Tests of rheumatic immune antibodies revealed that anti-CCP antibodies, anti-ENA antibodies, antinuclear antibodies, vasculitis antibody series, antiphospholipid antibody series, and rheumatoid arthritis antibody series were negative. Demyelinating disease examination of the central nervous system revealed that the anti-AQP4 antibody IgG, anti-MOG antibody IgG, and anti-MBP antibody IgG were negative. Paraneoplastic syndrome examination revealed that anti-Hu antibody IgG, anti-Yo antibody IgG, anti-Ri antibody IgG, anti-CV2 antibody IgG, anti-Ma2 antibody IgG, anti-amphiphysin antibody IgG, anti-ANNA-3 antibody IgG, anti-PCA-2IgG antibody, and anti-GAD antibody IgG were negative. Parasite panels of the liver, lungs, and brain showed that antibodies for liver fluke, lung fluke, hydatid, Toxoplasma gondii, cerebral cysticercus, sparganum, and *Schistosoma japonicum* were negative. In the peripheral blood smear (Fig. 1A, B), the morphology of the granulocytes, lymphocytes and platelets was basically normal. Mild anisocytosis was observed in some mature erythrocytes. Some monocytes had intracytoplasmic vacuoles. No nucleated erythrocytes, plasmodium, or microfilaria were observed. Bone marrow puncture revealed the following findings (Fig. 1C): active bone marrow hyperplasia; hyperplasia of granulocytes, erythrocytes and megakaryocytes; slightly high levels of plasma cells; and individual atypical lymphocytes. In the cerebrospinal fluid, pressure was 165 mm H_2_O, glucose was 3.09 ↓ mmol/L, protein was 0.757 ↑ g/L, globulin (qualitative) was positive (+), and cell count was 4/µL. Bacterial, *Mycobacterium tuberculosis* and fungal cultures were negative. Cytopathologic diagnosis of cerebrospinal fluid revealed visible individual lymphocytes in the smear. Electrocardiogram was normal. A noncontrast brain CT scan revealed no obvious abnormalities. CT of the nasopharynx, neck, chest and whole abdomen (pelvis included) revealed multiple small lymph nodes in the bilateral neck and groin and mild fatty liver; no definite abnormal signs observed on the noncontrast CT scan of the remaining regions of the nasopharynx, neck, chest, or whole abdomen. On transcranial Doppler, the blood flow velocity of the right vertebral artery was slightly decreased. Color ultrasound of the cervical vessels showed no abnormal echoes or images in the bilateral carotid or vertebral arteries. Color ultrasound of the superficial mass revealed a subcutaneous solid lesion behind the neck. Color ultrasound of extremity vessels showed no obviously abnormal echoes or images in the arteries or veins of the bilateral lower extremities. Heart color ultrasound revealed no significant abnormalities in the morphology, structure or valve mobility of the heart. No obvious abnormalities were observed in ventricular wall motion at rest. The left ventricle showed normal systolic function. Scrotum color ultrasound revealed abnormal echoes and images in the left testicle, and an inflammatory lesion was suspected. No abnormal echoes or images were observed in the right testicle. No abnormal echoes or images were noted in the bilateral epididymides. Left spermatic corditis was observed. There was no obvious abnormality in the right spermatic cord. On electromyography, incomplete damage to the right facial nerve, left facial nerve damage(manifestation during the recovery period of chronic lesions) and mild facial muscle synkinesis (left side) were observed. The distal motor and sensory conduction functions of the peripheral nerves of both lower extremities and the right upper extremity were normal or somewhat poor (sensory nerve action potential of the left superficial peroneal nerve was significantly decreased). The proximal motor conduction F wave was significantly impaired (the F wave of the tibial nerve of the right lower extremity was significantly slowed, with a markedly reduced occurrence rate) or slightly impaired (the F wave occurrence rate of the median and ulnar nerves of the right upper extremity was lower). H reflex examination of the left and right tibial nerves (soleus muscle) revealed no definite H reflex, indicating poor nerve conduction function at the proximal end of S1. The medial vastus muscle, tibialis anterior muscle, and gastrocnemius muscle of the right lower extremity and the left and right digit abductor muscles exhibited mild neurogenic lesions on electromyography, which were more significant in the terminal muscles. Several evoked potential tests were performed. Pattern reversal visual evoked potentials: The visual pathways of the eyes showed normal conduction function. Brainstem auditory evoked potential: there was slow conduction in the superior segment of the brainstem in the auditory pathway of the ears (III–V wave peak interval > I–III wave peak interval). Somatosensory evoked potential: The somatosensory pathway of both lower extremities showed normal conduction function upon stimulation of the medial malleolus segment of the tibial nerve of the lower extremities (no abnormalities in P40 and N9 latency). Brain + non-contrast MRA + spine and spinal cord MRI revealed the following: no abnormal signs on non-contrast MR scans or MRAs of the head; abnormal signals at T10 and T11, suggesting fat deposition; and mild backward herniation of the C4/5-C6/7 intervertebral disc, cervical spine degeneration, a high possibility of small hemangioma in C7, and backward herniation of the L4/5 intervertebral disc. Lymph node histopathology revealed high-grade B-cell lymphoma, which was consistent with double-hit lymphoma (Fig. 1D–G). Immunohistochemistry (IHC): CD3(−), CD5(−), CD20(+), CD79a(+), Bcl2(weakly+), Bc16(+), CD10(−), CD21(FDC+), Muml(partially +), Ki67(appr. 90%+), PAX5(+), CD23(FDC+), CyclinD1(−), Bc12(+/−), and MPO(−). In situ hybridization revealed EBERs(−). The fluorescence in situ hybridization results were as follows: IgH/Bcl2 *t*(14:18)(q32;q21) chromosomal translocation: negative; Bc16 gene translocation: positive; Myc(8;q24) chromosomal translocation: positive. The clonal gene rearrangement test for B-cell lymphoma was positive, and the clonal gene rearrangement test for T-cell lymphoma was negative.

The patient was diagnosed with high-grade B-cell lymphoma (with peripheral nerve damage as the initial manifestation) and was later transferred to a local oncology hospital for treatment. The treatment plan included chemotherapy, targeted drug therapy, biological therapy, immunotherapy, etc. The patient who was followed up for 1 and 3 years had stable conditions and was able to take care of himself, with an mRS score of 1. Five years after the initial diagnosis, the patient experienced recurrence and systemic metastasis of high-grade B-cell lymphoma, ultimately dying from multiple organ failure.

## 3. Discussion

### 3.1. Clinical characteristics of high-grade B-cell lymphoma manifesting as peripheral nerve injury

High-grade B-cell lymphomas are a group of highly aggressive lymphomas with morphological and genetic characteristics between those of diffuse large B-cell lymphoma and Burkitt lymphoma, with Myc and BCL2 and/or BCL6 rearrangements; this lymphoma often presents with aggressive behavior and involves the lymph nodes and extranodal organs (especially the bone marrow and nervous system).^[3–5]^ Lymphoma usually starts with unexplained fever, night sweats and weight loss. Patients with lymphoma have multiple enlarged lymph nodes throughout the body, possibly leading to central and peripheral nerve injury. The laboratory findings mainly revealed anemia, elevated lactate dehydrogenase levels and an increased erythrocyte sedimentation rate. Cerebrospinal fluid examination revealed elevated protein and increased lymphocyte counts, and the symptoms partially improved after hormone therapy.^[1,2,6]^ Lymphoma involvement in the central nervous system commonly occurs in the pia mater and midline structures (paraventricular, basal ganglia, corpus callosum, thalamus, brainstem, etc).^[1,6]^ Lymphoma involvement in the peripheral nervous system mainly manifests as multiple mononeuropathies of the extremities and impairment of multiple cranial nerves. Multiple mononeuropathies are more common than symmetric polyneuropathies and are typically characterized by subacute or chronic disease progression.^[6–8]^ Peripheral nerve injury caused by lymphoma can be divided according to pathological type as follows: peripheral nerve injury induced by direct infiltration of lymphoma cells; inflammatory demyelinating polyneuropathy; sensory axonal degeneration; vasculitic neuropathy; and paraproteinaemia-related neuropathy. The cause of nerve injury in most patients is peripheral nerve demyelination and/or nerve trunk distal axonal degeneration.^[7]^ In the early stages of cranial nerve lesions, the facial nerve, oculomotor nerve, trigeminal nerve and auditory nerve can be involved, and the optic nerve and abducens nerve are mildly involved.^[9,10]^ The cranial nerves most commonly invaded by head and neck tumors are widely distributed among the head and neck, facial nerve, trigeminal nerve, and oculomotor nerve.^[9]^ Sciatic nerve lesions are the most common type of mononeuropathy in the extremities.^[6,11]^ Immune dysfunction caused by lymphoma may also play a key role in peripheral nerve injury. Hormone immunomodulatory therapy is more or less effective, especially in the early stages, which contributes to the misdiagnosis of lymphoma nerve damage.^[7]^ Some patients with B-lymphoma even test positive for anti-ganglioside antibodies,^[12]^ which makes it more difficult to differentiate the disease from inflammatory demyelinating polyneuropathy. The patient’s facial nerve, oculomotor nerve, and auditory nerve were alternately involved multiple times, complicated with peripheral nerve injury of the lower extremities. The symptoms improved after hormone treatment, which is not consistent with the common clinical manifestations of other peripheral neuropathies. The patient had a history of hormone use 1 week before onset. Hormone use complicates the diagnosis of lymphoma; lymph node enlargement subsides due to the use of hormones, making it difficult to perform a biopsy and affecting the pathological results of lymphoma tissue biopsy. Therefore, we advised the patient to stop the use of hormones immediately after admission.

### 3.2. Characteristics of PET–CT-negative lymphoma

Lymphoma involving the peripheral nerves mostly manifests as B-lymphoma^[13]^ and rarely as a solitary mass. Most patients present with diffuse or solitary thickening of nerves/nerve roots in the form of mononeuropathy, radiculopathy/plexopathy or generalized neuropathy, similar to chronic inflammatory demyelinating polyradiculoneuropathy (CIDP) or Guillain–Barré syndrome.^[11]^ PET–CT is a useful adjunct method in the evaluation of lymphoma peripheral neuropathy, especially for patients with neurolymphomatosis. Its sensitivity can reach 83.3% to 100%.^[14]^ For patients with a known history of lymphoma, PET–CT plays a key role in the evaluation of therapeutic effects and the likelihood of recurrence.^[15]^ However, PET–CT-negative cases have also been reported.^[16]^ Neurological symptoms may precede lymphoma, and in the early stage of neurological symptoms, patients may be complicated with or without fever, lymph node enlargement, hepatosplenomegaly, or other kinds of organomegaly.^[6,11]^ PET–CT findings show increased metabolic activity in tumor lesions and increased 18F-fluorodeoxyglucose (FDG) uptake, which are not observed in benign nerve tumors.^[8]^ The use of glucocorticoids may also affect FDG uptake during PET–CT, resulting in false negative results.^[14]^ Intravascular lymphoma is a rare subtype of diffuse large B-cell lymphoma that affects mainly middle-aged and elderly people. It is limited to the lumen of small- and medium-sized blood vessels and can involve the peripheral nervous system. Of the cranial nerves, the facial nerve and auditory nerve are often involved. PET–CT results are typically negative.^[6]^ The patient’s initial symptom was facioplegia, and color ultrasound indicated testicular enlargement. PET–CT examination performed 6 months before the diagnosis of lymphoma revealed no signs of lymphoma-induced peripheral nerve injury. This absence of signs was considered a result of inflammatory demyelinating immune dysfunction or paraneoplastic syndrome prior to the occurrence of lymphoma, coupled with multiple doses of hormones, which may affect FDG uptake. Nevertheless, peripheral nerve MRI and PET–CT examinations are highly important for the early diagnosis of peripheral nerve injury caused by malignant tumors such as lymphoma.

### 3.3. Differential diagnosis of high-grade B-cell lymphoma manifested as peripheral nerve injury

The patient was a middle-aged male, and the disease manifested as recurrent alternating peripheral facial paralysis, left oculomotor paralysis and symmetric fatigue in both lower extremities, complicated with night sweats, emaciation, right hip pain and unsteady walking. Physical examination revealed binocular nystagmus (+), a right eyelash sign (+), a shallow right nasolabial fold and loss of the tendon reflex of both lower limbs. The patient had a history of chronic left epididymo-orchitis, and PET–CT performed at another hospital revealed streak-shaped hypermetabolic foci in the bilateral scrota and multiple enlarged lymph nodes in the bilateral submandibular, bilateral substernocleidomastoid muscle and bilateral inguinal regions. Hormone therapy was effective. Given the characteristics of mildly elevated cerebrospinal fluid protein levels, lymph node histopathology, Myc and BCL-6 rearrangement detected by standard cytogenetics, and weak Bcl-2 positivity, the disease is consistent with a diagnosis of high-grade B-cell lymphoma. Differentiation from the following diseases was considered: CIDP: CIDP is characterized by chronic progression or relapsing-remitting with 8 + weeks of symptom progression. The main clinical manifestation is different degrees of limb weakness, mostly symmetrical and occasionally asymmetrical, involving both the proximal and distal limbs. The tendon reflex of limbs is reduced or absent, accompanied by deep and superficial paresthesia, and impairment of multiple cranial nerves can occur concurrently. Albuminocytologic dissociation can be detected in cerebrospinal fluid; the protein level is between 0.75 and 2.00 g/L and even higher in some patients. Electrophysiological examination indicates a slower peripheral nerve conduction velocity, conduction block or abnormal wave dispersion. Glucocorticoid treatment is effective.^[17,18]^ However, in addition to peripheral neuropathy, this patient also experienced recurrent testicular enlargement, multiple swollen lymph nodes throughout the body, and emaciation and night sweats over recent days, which are not explained by CIDP. Polyneuropathy, organomegaly, endocrinopathy, monoclonal paraproteinaemia, and skin changes (POEMS) syndrome: Progressive multiple peripheral neuropathy can be the first symptom of POEMS, manifested as symmetrical motor and sensory impairments of the limbs, with the lower limbs being heavier than the upper limbs and the distal end being heavier than the proximal end. These impairments are typically accompanied by foot drop, amyotrophy and impaired or absent knee reflexes, hepatosplenomegaly or other kinds of organomegaly. Some patients may have enlarged lymph nodes, which are often complicated with monoclonal plasma cell disorders. Patients with enlargement of the lymph nodes may also be complicated with Castleman’s disease or lymphoma,^[6]^ and some patients may suffer from endocrine dysfunction, such as gynecomastia. Most patients have normal liver function. Some patients exhibit skin pigmentation and respond to hormonotherapy.^[19]^ However, the results of serum protein electrophoresis, Bence Jones protein analysis, bone marrow aspiration and other examinations do not support the presence of monoclonal plasma cell disorders in this patient; thus, POEMS can be excluded from the diagnosis. Castleman’s disease: Castleman’s disease is a rare lymph node proliferative disease. Unicentric Castleman’s disease mostly manifests as large cervical or abdominal lymph nodes and is typically not complicated by peripheral neuropathy. Multicentric Castleman’s disease manifests as enlargement of the lymph nodes and is typically accompanied by multisystem involvement and peripheral neuropathy. A few patients may suffer from fever, hepatosplenomegaly, anemia, thrombocytopenia, hypoalbuminemia, and elevated immunoglobulins. Approximately 20%-30% of cases may be complicated with Kaposi’s sarcoma or B lymphoma, amyloidosis or Sjogren’s syndrome during the disease course. When complicated with other diseases or complications, the disease may appear to be POEMS syndrome. In addition, multicentric Castleman’s disease often has an aggressive course and is typically accompanied by human immunodeficiency virus or herpes simplex virus 8 infection.^[13,20]^ However, this patient had no prodromal history of human immunodeficiency virus or herpes simplex virus 8 infection, and the results of the lymph node biopsy did not support the diagnosis of lymph node proliferative disease; thus, this condition can be ruled out. Bickerstaff brainstem encephalitis (BBE): BBE is a rare autoimmune disease that manifests mainly as external ophthalmoplegia, ataxia, impaired consciousness, long pyramid signs, and sensory disorders. It is usually characterized by acute or subacute onset and impairment of multiple cranial nerves and conduction bundle functions. Most patients are complicated with a prodromal history of infection and respond to hormonotherapy or immunotherapy. The clinical manifestations of BBE may overlap with those of Fisher syndrome and Guillain–Barré syndrome, and BBE patients can be positive for anti-GQ1b antibodies according to serological tests^[21,22]^; however, these patients rarely suffer from multiple enlarged lymph nodes throughout the body and swollen testes, so BBE can be ruled out in this patient. Peripheral neuropathy caused by poisoning, metabolic diseases or connective tissue diseases: Poisoning; metabolic diseases; frequent administration of isoniazide, macrodantin and other medicines; a history of toxic exposure^[23,24]^; and diagnosed diabetes, uremia, hypothyroidism,^[25–27]^ systemic lupus erythematosus, Behcet’s syndrome, Sjogren’s syndrome and other systemic autoimmune diseases may involve the brainstem, spinal cord and peripheral nerves.^[28–30]^ However, patients with these diseases generally have clinical manifestations and serological test results related to organ injury. For example, systemic lupus erythematosus and Behcet’s syndrome patients usually have skin and mucosal damage, and Sjogren’s syndrome patients often have symptoms such as dry mouth and dry eyes, with positive serum SSA and SSB antibodies, which are generally not accompanied by organomegaly. Paraneoplastic syndrome: Symptoms of sensory impairment are more obvious in paraneoplastic syndrome patients, manifesting as decreased or absent deep/superficial sensations, and impairment of multiple cranial nerves or sensory ataxia can occur as a result of cancer-induced nonmetastatic peripheral nerve injury.^[31]^ This patient had a history of repeated abnormal space-occupying lesions in the testicles, and the possibility of reproductive system tumors, which can be differentiated mainly through a comprehensive examination of cancer, needs to be ruled out.

Although this study provides a detailed report on a case of high-grade B-cell lymphoma manifesting as peripheral nerve injury, the study still has notable limitations. Firstly, as a single case report, the universality and reliability of its conclusions indeed require further verification, as the uniqueness of a single case may not fully represent the common characteristics of the entire patient population. Secondly, due to the lack of sufficient control group data, it is difficult to accurately determine whether the peripheral nerve injury is indeed directly caused by high-grade B-cell lymphoma or may be influenced by other potential diseases or factors. Additionally, the report does not provide detailed pathological analysis and long-term follow-up results, which limits our in-depth understanding of disease progression, pathological mechanisms, and patient prognosis. Therefore, future research needs to more thoroughly explore this disease manifestation by early identification of more cases and conducting detailed studies to further verify and deepen our understanding of the relationship between high-grade B-cell lymphoma and peripheral nerve injury.

## 4. Conclusions

Lymphoma manifests mainly as progressive impairment of multiple cranial nerves or recurrent alternating peripheral nerve injury, is complicated with elevated protein concentrations in cerebrospinal fluid, and can be easily misdiagnosed as CIDP. In patients with poor therapeutic effects after repeated treatment or fever accompanied by organomegaly, physicians must be highly aware of the possibility of lymphoma, and close clinical follow-up is needed. Lymph node biopsy for high-grade B-cell lymphoma performed as early as possible helps detect lymphoma in the early stage and aids in the timely selection of reasonable chemotherapy or targeted therapy.

## Acknowledgments

We would like to express our gratitude to the patient’s family members for granting permission to use the patient’s clinical data in this paper and for the publication of this research.

## Author contributions

**Conceptualization:** Bo Liu, Han Luo.

**Data curation:** Bo Liu, Han Luo, Shanshan Li.

**Formal analysis:** Bo Liu, Han Luo.

**Funding acquisition:** Bo Liu, Han Luo.

**Investigation:** Bo Liu, Han Luo, Shanshan Li.

**Methodology:** Bo Liu, Han Luo.

**Project administration:** Bo Liu, Han Luo, Shanshan Li.

**Resources:** Bo Liu, Han Luo.

**Software:** Bo Liu, Han Luo.

**Supervision:** Bo Liu, Han Luo.

**Validation:** Bo Liu, Han Luo.

**Visualization:** Bo Liu, Han Luo, Shanshan Li.

**Writing – original draft:** Han Luo.

**Writing – review & editing:** Bo Liu.
